# Efficacy of vagus nerve stimulator during transition to adulthood in patients with treatment‐resistant epilepsy

**DOI:** 10.1002/epd2.70133

**Published:** 2026-01-19

**Authors:** Jonadab dos Santos Silva, Shaylla Villas Boas, Henrique Januzzelli Pires do Prado, Daniela Fontes Bezerra, Isabella D’Andrea Meira

**Affiliations:** ^1^ Department of Neurology Yale School of Medicine New Haven Connecticut USA; ^2^ Epilepsy Center Paulo Niemeyer State Brain Institute Rio de Janeiro Brazil; ^3^ Fluminense Federal University, Postgraduate Program in Neurology and Neuroscience Niterói Brazil; ^4^ Department of Epilepsy Faculdade de Medicina do ABC São Paulo Brazil

**Keywords:** adolescence, atonic seizure (drop attack), clonic seizure, generalized tonic‐clonic seizure, Lennox–Gastaut syndrome, puberty, vagus nerve stimulation

## Abstract

**Objective:**

To evaluate the efficacy of vagus nerve stimulation (VNS) as an adjunctive treatment in pediatric patients with treatment‐resistant epilepsy during the transition to adolescence.

**Methods:**

We performed a retrospective cohort study of 30 children (ages 2–18 years) with medication‐resistant epilepsy who underwent VNS implantation between January 2019 and January 2023 at a tertiary epilepsy center. Clinical data included demographics, epilepsy etiology and syndrome, age at implantation, seizure frequency and severity (McHugh classification), number of antiseizure medications (ASM), magnet use, and EEG findings. Outcomes were assessed at the last available follow‐up, with a minimum duration of 12 months post‐implantation. Statistical analyses comprised chi‐squared or Fisher's exact tests, Spearman rank correlation, and logistic regression; significance was set at *p* < 0.05.

**Results:**

At the last follow‐up, 20 of 30 patients (66.7%) achieved ≥50% reduction in seizure frequency, and 73% of patients experienced a significant decrease in seizure severity. Status epilepticus (SE) incidence fell from 67% pre‐VNS to 17% post‐VNS (*p* = 0.024). The number of ASMs decreased in 57% of patients. Successful magnet activation strongly predicted responder status (OR 10.8, 95% CI 1.2–95.4; *p* = 0.024). Among females, 12 of 16 (75%) experienced a transient seizure worsening around menarche. Earlier VNS implantation correlated with better seizure reduction (ρ = −0.43; *p* = 0.015). EEG improvement—defined as reduced interictal epileptiform activity or background normalization—was observed in 23% of patients, predominantly in males and pre‐menarche females.

**Significance:**

VNS is an effective adjunctive therapy in pediatric treatment‐resistant epilepsy, yielding substantial reductions in seizure frequency and severity, lowering status epilepticus risk, and simplifying medication regimens. Pubertal status and magnet responsiveness modulate outcomes. Early VNS intervention and proactive management during adolescence may optimize therapeutic benefit.

**Abbreviations**
ASM: antiseizure medication
CI: confidence interval
EEG: electroencephalography
LGS: Lennox–Gastaut syndrome
OR: odds ratio
SE: status epilepticus
VNS: vagus nerve stimulation

**Key points**

- Two‐thirds of pediatric treatment‐resistant epilepsy patients achieved ≥50% seizure reduction with VNS.
- VNS reduced convulsive status epilepticus incidence from 67% preimplant to 17% postimplant.
- Over 50% of patients reduced antiseizure medications following VNS, simplifying adolescent treatment.
- Transient seizure exacerbation around menarche occurred despite VNS, emphasizing proactive pubertal management.
- Acute seizure interruption with the VNS magnet predicted long‐term seizure reduction.

## 1 INTRODUCTION

Adolescence is a key period in epilepsy therapy, marked by hormonal changes, psychosocial stressors, and the need for drug adjustments.^1^, ^2^ Puberty can have a considerable impact on seizure patterns; increased seizure frequency is common, most likely due to shifting hormone levels,^2^ and some epilepsy syndromes even emerge during this period.^2^, ^3^ The effect is most noticeable in females, who often have increased seizure activity around menstruation due to estrogen and progesterone fluctuations.^2^

Treatment‐resistant epilepsy in adolescents is usually associated with cognitive impairment and mental comorbidities, including depression.^4^ Lifestyle issues such as sleep deprivation, irregular medication adherence, and substance abuse impede treatment.^1^ Simplifying polytherapy and increasing seizure control during the transition to adulthood are thus critical objectives.^1^

Vagus nerve stimulation (VNS) is an approved therapy for treatment‐resistant seizures in individuals aged 4 and up.^5^, ^6^ VNS regulates numerous brain circuits, increasing the seizure threshold and providing a multimodal approach to seizure control.^7^ Early VNS treatments can reduce seizure frequency in infants with treatment‐resistant epilepsy by 50–60%.^5^, ^6^, ^7^ This efficacy extends to severe epileptic disorders such as Lennox–Gastaut syndrome (LGS) and Dravet syndrome, with reported response rates of approximately 55% and 53%, respectively.^8^ Beyond seizure frequency reduction, VNS frequently improves seizure severity, postictal recovery, and even cognitive performance, making it an appealing treatment option for pediatric patients.^9^ These numerous advantages make VNS an intriguing alternative in the pediatric age group. While the general efficacy of VNS in children is well established, there is a notable gap in the literature regarding its performance during the tumultuous period of adolescent transition, where hormonal, psychosocial, and adherence‐related factors create unique clinical challenges.

Given the complexities of managing epilepsy during adolescence, this study investigates VNS efficacy in this vulnerable population. We examine the influence of patient sex, pubertal status, epilepsy etiology/syndrome, and clinical features on VNS response, focusing on seizure reduction, changes in seizure severity, number of antiseizure medications (ASM), and EEG findings. We aim to identify factors predictive of positive outcomes, ultimately guiding more effective management strategies to improve seizure control and overall quality of life for young people with treatment‐resistant epilepsy.

## 2 METHODS

### 2.1 Study design and participants

We conducted a retrospective cohort study of pediatric patients with treatment‐resistant epilepsy who were treated with VNS at our tertiary epilepsy center. Data were collected by review of medical records from January 2019 to January 2023, and the study was approved by the institutional Research Ethics Committee. Inclusion criteria were patients of either sex with treatment‐resistant epilepsy (failure of ≥2 appropriate antiseizure medications) who received VNS implantation between 2 and 18 years of age, with a post‐implant follow‐up duration of at least 12 months. We excluded patients who underwent another epilepsy surgery after VNS implantation (to isolate the effect of VNS), those with < 12 months of follow‐up, and cases with substantially incomplete data.

### 2.2 Study population and selection

A total of 55 patients with VNS implantation were initially randomly identified. From this cohort, patients were excluded for the following reasons: subsequent resective or disconnective epilepsy surgery after VNS implantation (*n* = 3), age > 18 years at the time of implant (*n* = 4), follow‐up duration of < 12 months (*n* = 5), lack of regular ambulatory follow‐up at our center during the study period (*n* = 6), and incomplete medical records that precluded the classification of seizure response according to the McHugh et al. criteria (*n* = 7). This selection process resulted in a final study sample of 30 patients who met all inclusion and exclusion criteria.

### 2.3 Data collection

Patient records provided demographic and clinical data. Age and gender were demographic variables. Epilepsy onset and VNS implantation ages were recorded, and we computed pre‐VNS epilepsy duration. Female patients' age at menarche and whether they had menarche at VNS implantation were recorded. We documented if a female patient's seizures worsened around menarche. Analytically, female pubertal status was a categorical variable. Epilepsy etiology and syndromes were recorded. Lennox–Gastaut syndrome (LGS), Dravet syndrome, and myoclonic‐astatic epilepsy (Doose syndrome) were also found clinically. The most common seizure types were generalized‐onset, focal‐onset, both/mixed, or unknown‐onset, while certain disorders had generalized tonic–atonic (“drop”) seizures.

We noted any prior epilepsy surgeries before VNS and whether the patient had a history of status epilepticus before VNS. The use of concurrent antiseizure medications (ASMs) and the number of ASMs at the time of VNS implant and last follow‐up were documented. From this, we determined if the number of medications decreased following VNS. A history of status epilepticus, defined as a convulsive seizure lasting longer than 5 min or a series of seizures without recovery requiring hospital admission, was recorded.

### 2.4 Outcomes and follow‐up

Baseline seizure frequency was determined from clinical records from the 3‐month period preceding VNS implantation. The primary outcome assessment was conducted at the last available follow‐up appointment for each patient, with a required minimum follow‐up of 12 months. The McHugh et al.^10^ classification of VNS response categorizes seizure frequency outcomes as no improvement, < 50% reduction, 50–80% reduction, and 80–100% reduction (almost complete control). In some analyses, patients were categorized as responders (≥50% seizure frequency decrease) or non‐responders (≤50% reduction). Changes in seizure intensity were examined alongside frequency. Clinical notes showed if VNS improved seizure severity and postictal recovery. Patient or caregiver reports that swiping the magnet during seizure start interrupted or shortened seizures were additionally noted. Although data on the use of the automatic stimulation (AutoStim) feature were not systematically collected, all families received uniform training on magnet use at device activation.

EEG alterations during VNS therapy were also assessed. All patients obtained regular or video‐EEGs before and after VNS implantation. EEG recordings showed interictal epileptiform abnormalities before and after ≥1 year of VNS, including widespread slowness, spikes, and multifocal discharges. EEG improvement was determined by the consensus of two independent neurophysiologists blinded to clinical response. Pre‐implantation and latest follow‐up EEGs (minimum 30‐minute recordings) were compared. Improvement was defined as a ≥ 50% reduction in the quantity of interictal epileptiform discharges.

### 2.5 Statistical analysis

All data were analyzed utilizing SPSS 27 (IBM Corp., Armonk, NY). Continuous variables are expressed as mean ± standard deviation (SD) or median and range, if applicable, whereas categorical variables are presented as counts and percentages. We conducted bivariate analyses concentrating on categorical predictors and outcomes. Relationships between categorical factors and outcomes were evaluated using the chi‐squared test or Fisher's exact test, accompanied by odds ratios (OR) and 95% confidence intervals (CIs) to quantify effect magnitude. For ordinal or continuous variables that violated parametric assumptions, we used the Spearman rank correlation to investigate associations. A two‐tailed *p*‐value < 0.05 was deemed statistically significant for all analyses.

## 3 RESULTS

### 3.1 Patient characteristics

Included were 30 patients (16 females, 53.3%). The median age at last follow‐up was 16.0 (range 9–29). The mean age at VNS implantation was 10.3 ± 4.1 years, while the median age for epilepsy onset was 1 year (range 0–12). On average, VNS was implanted 8 years post‐seizure onset (mean epilepsy duration pre‐VNS: 8.0 ± 4.4 years). Median follow‐up interval was 2.5 years (range 1–16 years). Table 1 shows the cohort's baseline clinical characteristics. In 13 cases (43.3%), structural etiologies caused epilepsy. This was followed by structural‐genetic factors in 7 (23.3%), genetic causes in 5 (16.7%), and unknown causes in 5 (16.7%). Nine (30%) had Lennox–Gastaut syndrome, one (3.3%) had Dravet syndrome, and two (6.7%) had myoclonic‐astatic epilepsy. The remaining patients (*n* = 18) had medication‐resistant epilepsy not classified under a specific syndrome. Six (20%) individuals had epilepsy surgery before VNS: four resective and two callosotomy. Pre‐VNS status epilepticus occurred in 19 (63.3%) patients. During VNS implantation, all patients took a median of 4 (range 2–5) antiseizure medicines. Each female patient had reached menarche, with a mean age of 12.1 ± 1.8 years. Twelve (75%) of 16 female patients' caregivers reported worsening seizures after menarche. Patients who received VNS at menarche or post‐menarche had seizures intensifying around puberty. All seven female patients who experienced menarche during VNS therapy reported a transient increase in seizure frequency or severity, compared to 5 of 9 (55.6%) who had VNS implanted post‐menarche, indicating a trend toward worsening post‐menarche seizures (OR 11.20, 95% CI: 0.48–262.94, *p* = 0.088).

**TABLE 1:** Cohort characteristics.

*N*	30
Female sex, *n* (%)	16 (53.3)
Age at last follow‐up, years	16 (9–29)
Age at epilepsy onset, years	1 (0–12)
Age at VNS implantation, years	10.3 ± 4.1
Epilepsy duration before VNS, years	8.0 ± 4.4
Number of ASMs at VNS implant	4 (2–5)
Number of ASMs at last follow‐up	3 (2–5)
Mean age at menarche (females only), years	12.1 ± 1.8
Etiology of epilepsy	
Structural	13 (43.3)
Genetic	5 (16.7)
Structural + genetic	7 (23.3)
Unknown	5 (16.7)
Epilepsy syndrome	
Lennox–Gastaut syndrome	9 (30.0)
Dravet syndrome	1 (3.3)
Myoclonic‐astatic epilepsy	2 (6.7)
Other	18 (60.0)
Prior epilepsy surgery (pre‐VNS)	6 (20.0)
Resective surgery	4 (13.3)
Callosotomy	2 (6.7)
History of status epilepticus	19 (63.3)

*Note*: Data are presented as mean ± standard deviation (SD), median (range), or number (percentage), as appropriate.

Abbreviations: ASM, antiseizure medication; LGS, Lennox–Gastaut syndrome; VNS, vagus nerve stimulation.

### 3.2 Seizure frequency reduction with VNS

**FIGURE 1:**
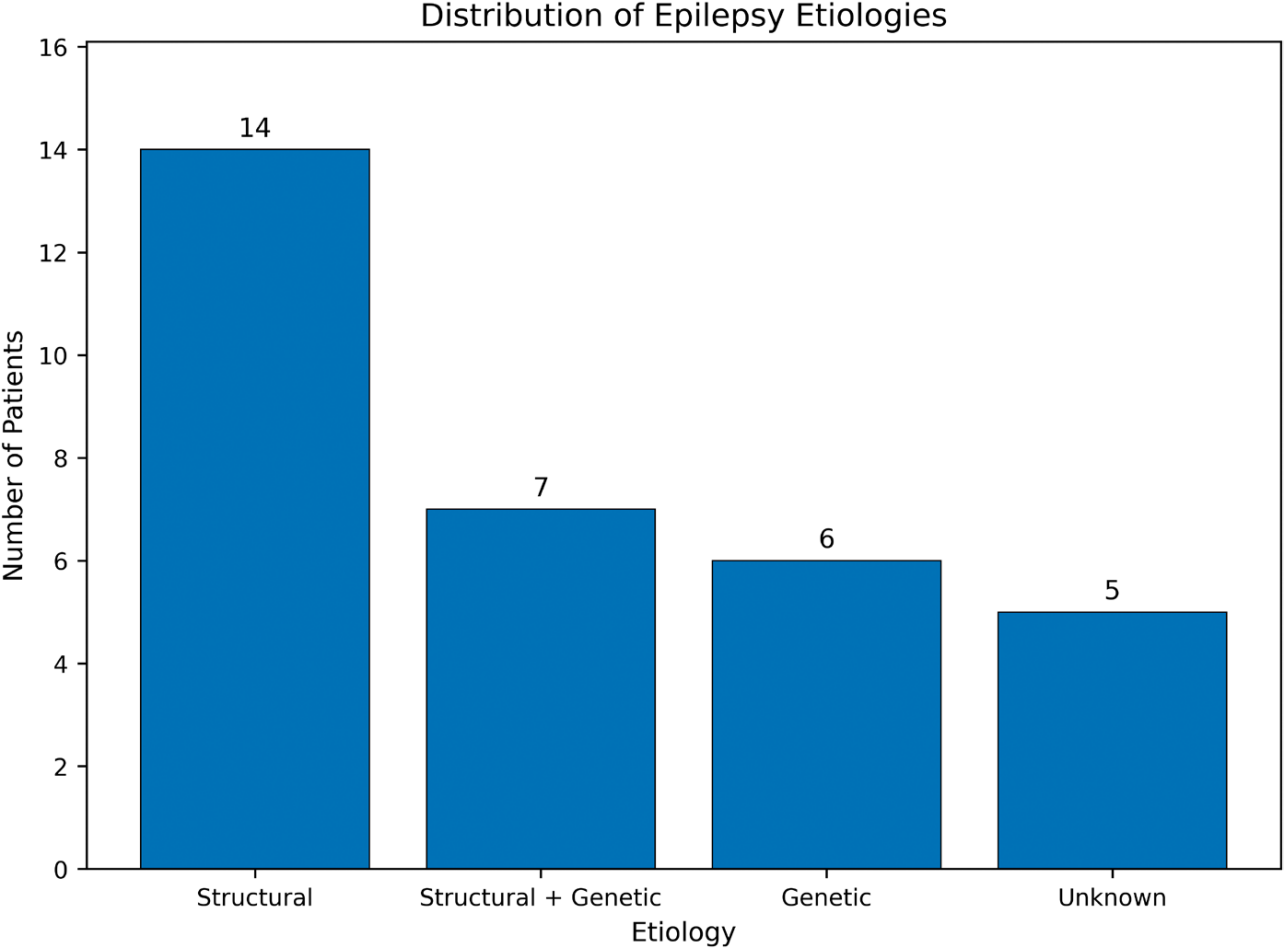
Distribution of epilepsy etiologies. Bar chart representing the number of patients with each major epilepsy etiology in our cohort. Etiologies were classified as “Structural,” “Structural + Genetic,” “Genetic,” or “Unknown.” Structural etiologies (*n* = 14) were most common, followed by combined structural–genetic (*n* = 7), purely genetic (*n* = 6), and unknown (*n* = 5).

**FIGURE 2:**
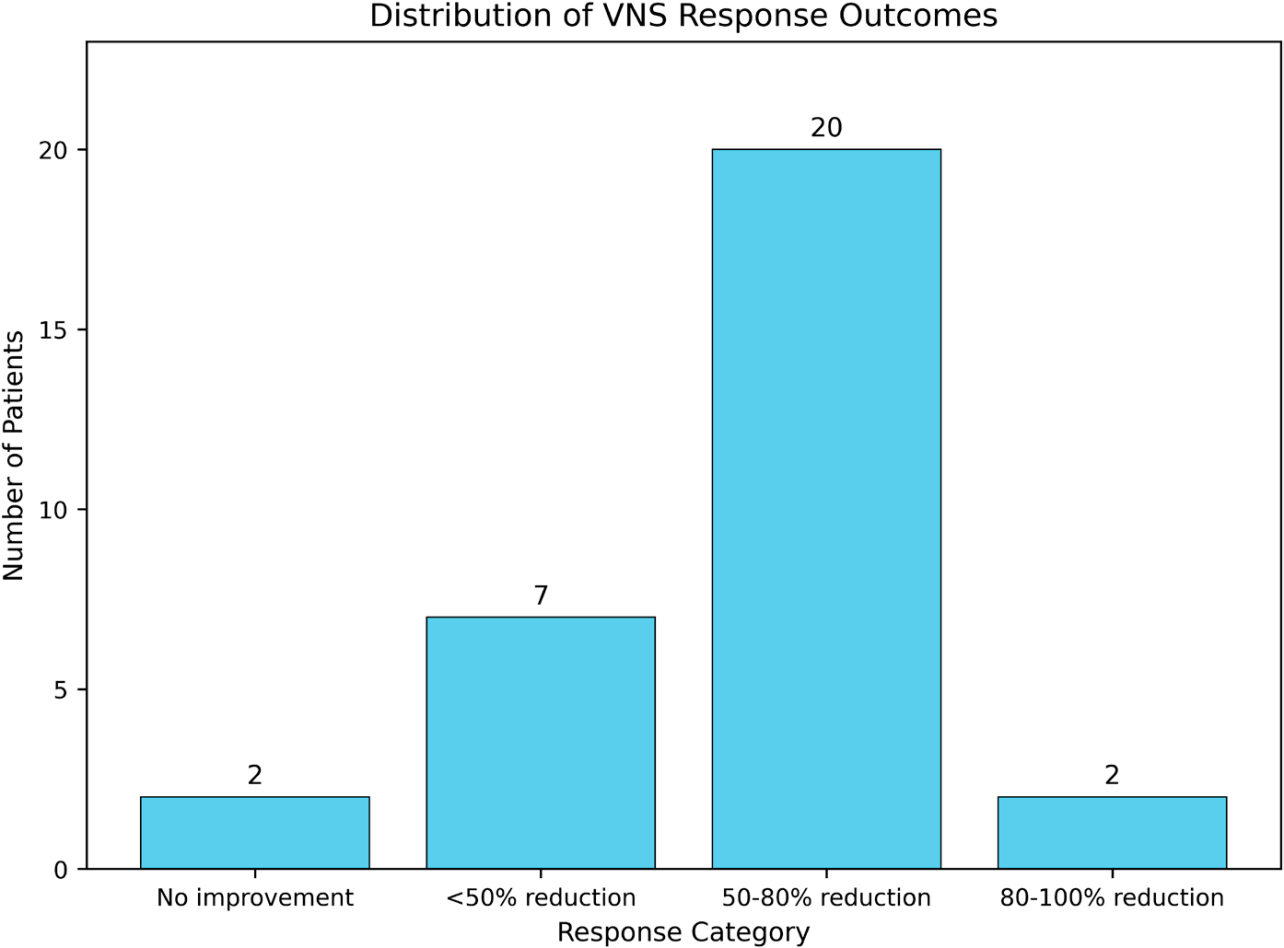
Distribution of VNS response outcomes. The bar chart depicts the number of patients in each VNS response category according to the McHugh classification. “No improvement” corresponds to no seizure reduction, “< 50% reduction” indicates a moderate or minor improvement, “50–80% reduction” indicates marked improvement, and “80–100% reduction” indicates near‐complete seizure control. Among our 30 patients, two showed no improvement, seven had < 50% reduction, twenty had 50–80% reduction, and two had 80–100% reduction.

Twenty of 30 patients (66.7%) showed a ≥ 50% reduction in seizure frequency at the last follow‐up visit of VNS therapy, indicating a therapeutic response. Eighteen (60%) had a 50–80% seizure reduction, while two (6.7%) had an 80–100% reduction, indicating near‐complete seizure control. Eight patients (26.7%) had a < 50% decrease in seizure frequency, whereas two (6.7%) showed minimal improvement or worsening. Thus, 10 patients with seizure reductions below 50% were non‐responders. Response distribution is shown in Figure 1. As shown below, partial responders often improved in other areas but did not eliminate seizures. Clinical records show that most responders took 6–12 months after VNS implantation to achieve a sustained > 50% seizure reduction. VNS efficacy did not affect seizure frequency by gender (Figure 2).

**FIGURE 3:**
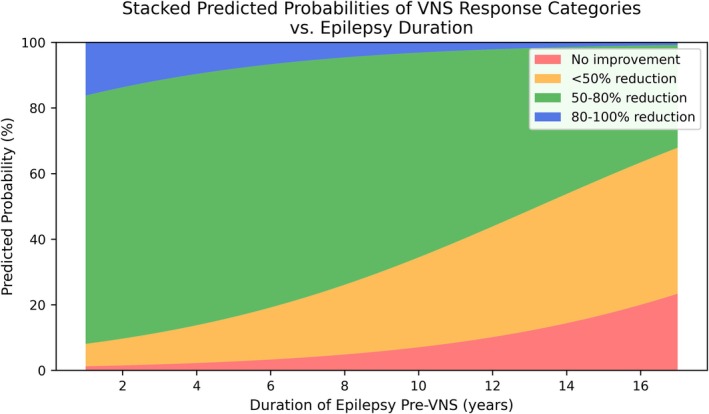
Stacked area chart of predicted probabilities (in percentages) for the four VNS response categories (no improvement, < 50% reduction, 50–80% reduction, or 80–100% reduction) across increasing epilepsy duration, based on the ordinal logistic regression model. At each value on the x‐axis, the colored regions sum to 100%, illustrating how the likelihood of higher seizure reduction (green and blue areas) decreases as epilepsy duration grows, while lower improvement (red and yellow areas) becomes more likely.

**FIGURE 4:**
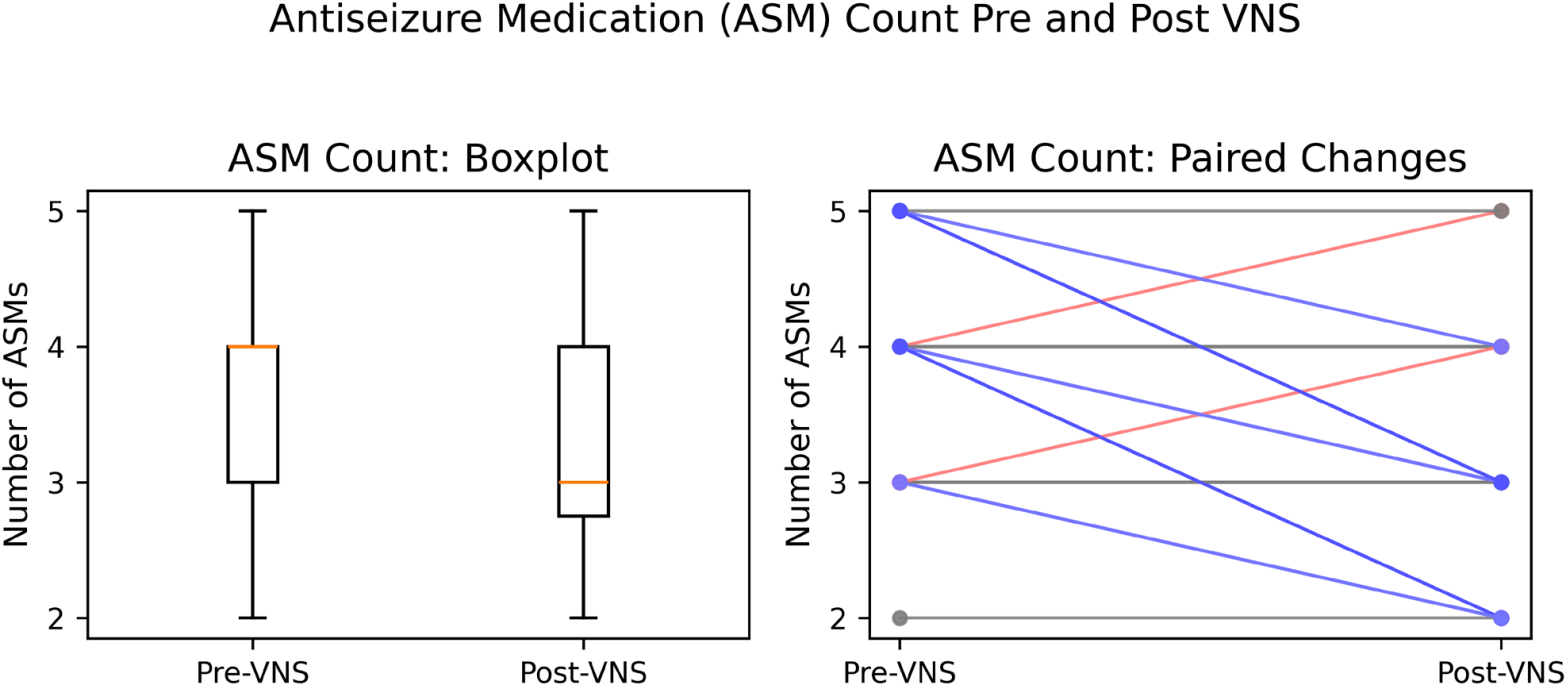
Antiseizure medication (ASM) count pre‐ and post‐VNS. The left panel (boxplot) displays the distribution of the number of antiseizure medications (ASMs) each patient was taking before (pre‐VNS) versus after (post‐VNS) vagus nerve stimulation. The right panel shows paired lines for each patient, illustrating individual changes in ASM count. Most patients demonstrated a decrease in the number of ASM (blue lines; red representing increase), reflecting the potential of VNS to simplify treatment regimens.

The study explored VNS age and timing effects on outcomes. For each year of uncontrolled epilepsy before VNS implantation, the odds of being in a lower response category increased by 22% (OR = 1.22, 95% CI = 1.01–1.47, *p* = 0.034), revealing that patients with longer periods of uncontrolled epilepsy were less likely to reduce seizures. Figure 3 demonstrates the output of the ordinal logistic regression model, illustrating the predicted probability of different VNS response outcomes based on the duration of epilepsy before implantation. The stacked colored bands represent the four response categories, from no improvement (red) to an 80–100% seizure reduction (blue). As the duration of epilepsy increases along the x‐axis, the model predicts a clear shift in probabilities: the areas corresponding to favorable responses (green and blue) diminish, while the areas for poor outcomes (orange and red) expand. This demonstrates that a longer history of uncontrolled epilepsy before VNS is associated with a lower likelihood of achieving significant seizure reduction (Figure 4).

### 3.3 Epilepsy etiology and syndromes

VNS was effective across different epilepsy etiologies, with no differences in responder rates by etiology. Patients with structural etiologies had a responder rate of 78.6% (11/14) compared to 61.1% (11/18) in those with non‐structural etiologies (OR = 2.33, 95% CI = 0.45–12.0). Importantly, no etiology or syndrome categorically precluded a response to VNS.

### 3.4 Seizure severity, magnet effect, and status outcomes

VNS reduces seizure frequency, severity, and recovery. Caregivers for 73.3% (22/30) of patients reported a substantial reduction in seizure severity and/or improvement in postictal recovery. Shorter seizure duration, less severe convulsions, and speedier recovery in youngsters were improvements. The subjective reduction in seizure intensity correlated with the overall frequency response. Only 2 of those reporting decreased seizure severity had a frequency reduction of < 50%.

Out of eight patients with unimproved seizures, 6 were non‐responders (OR = 40, 95% CI = 4.5–1775), indicating that lowered seizure severity was 40 times more likely to achieve a ≥ 50% drop in frequency. Seizure severity rarely improves without frequency reduction in clinical practice. In two cases, patients still had frequent seizures, but they were less severe after VNS.

Another important feature was magnet‐activated stimulation. When swiping the magnet at seizure start, 13 (43.3%) individuals had seizures terminated or reduced in severity. Families used the magnet during auras or seizures, with some reporting that it stopped them. Twelve of 13 patients with acute magnet benefit responded to VNS, compared to 10 of 17 without (OR = 10.8, 95% CI = 1.2–95.4, *p* = 0.024). Patients who stopped seizures with the magnet had a 92% chance of a 50% or more long‐term seizure reduction, compared to 59% without the magnet.

VNS therapy affected status epilepticus frequency. Twenty patients had intermittent status epilepticus before VNS. Only five patients (16.7%) suffered status epilepticus after VNS implantation. VNS did not cause status epilepticus in 25/30 (83.3%) patients. One of the five patients with post‐VNS status events satisfied the responder criteria, while four were non‐responders with numerous seizure types. This suggested a dismal prognosis. However, 20 of 25 (80%) people without post‐VNS status epilepticus responded (OR 0.07 for response if post‐VNS status epilepticus occurred, 95% CI = 0.004–0.9, *p* = 0.024). Patients without status epilepticus using VNS had a better chance of seizure control. After VNS, none of the four patients who had resective epilepsy surgery developed status epilepticus. However, 5 of 26 patients without prior surgery suffered status epilepticus post‐VNS (19.2%), with no significant difference.

### 3.5 Antiseizure medication use

Antiseizure medications (ASMs) per patient in our group decreased significantly after VNS therapy. Patients took a median of 4 ASMs (range 2–5) before VNS implantation. After one year of VNS, the median ASM count was 3, ranging from 2–5. We found that 17 patients (56.7%) reduced their antiepileptic drug (ASM) dosage, 13 (43.3%) did not adjust their prescription, and 2 were required to increase it. More than half of the cohort had a significant therapeutic load reduction (*Z* = 3.30, *p* < 0.001). Reducing drugs often improved clinical outcomes; 11 of 17 responders did so, although some nonresponders did so with careful observation. Lennox–Gastaut syndrome patients were less likely to reduce their drug burden than those without LGS: only 2 of 9 LGS patients (22.2%) reduced their ASMs, compared to 15 of 21 (71.4%) of those without LGS (OR = 0.11, 95% CI = 0.02–0.72, *p* = 0.020). There was no correlation between medication reduction and ≥ 50% response, as some non‐responders lowered prescriptions and others did not. Many clinical improvements were linked to therapeutic simplicity.

### 3.6 EEG findings

Serial EEGs measured VNS‐related neurophysiological changes objectively. Nearly all patients had abnormal baseline EEGs. Dilute slowness was found in 28 of 30 individuals, focal epileptiform discharges in 10, multifocal discharges in 12, and generalized spike‐and‐wave or polyspike bursts in 21, especially in those with generalized epilepsy syndromes. Seven patients (23.3%) showed considerable EEG improvement after one year of VNS therapy, with zero or fewer epileptiform abnormalities. The majority (77%) showed no meaningful change in EEG despite clinical enhancements, and EEG improvement did not correlate with clinical responder status.

A pubertal status‐EEG finding was noted. All seven EEG‐improved patients were male or prepubescent female. Before menarche, the two youngest girls had significant EEG improvements after VNS, while none of the post‐menarche girls did (14 post‐pubertal girls had consistently abnormal EEGs). We found that pre‐ and post‐menarche females had significantly different EEG improvement rates (100% vs. 12.5%, *p* = 0.04) due to pubertal development and the brain's electrophysiological response to VNS. Patients with structural etiologies showed EEG improvements. In 12 patients with initial multifocal spikes, 5 of 7 with structural etiology lost spikes after VNS, while only 1 of 5 with nonstructural etiology did (OR = 5.0, 95% CI = 0.3–77.1, *p* = 0.15). Following VNS, diffuse background slowing decreased in 4 individuals, but this did not correlate with clinical outcomes.

## 4 DISCUSSION

### 4.1 Efficacy of VNS in pediatric treatment‐resistant epilepsy

This study reveals the significant potential of VNS as an adjunctive therapy for treatment‐resistant epilepsy in pediatric patients. Our findings demonstrate a robust responder rate (≥ 50% seizure reduction) of approximately 67%, exceeding, albeit slightly, the 50–60% range typically reported in larger pediatric studies.^7^ This coincides with the American Academy of Neurology's evidence‐based study, which found 55% responders in 470 children, including LGS. VNS reduced seizures in two‐thirds of our pediatric population, demonstrating its efficacy. While VNS cannot eliminate seizures, the statistics show reductions in seizure frequency and severity. In our cohort, 73% of patients saw qualitative benefits such as shorter or less severe seizures without meeting the 50% frequency reduction threshold for responders. This emphasizes the limitations of traditional responder definitions in capturing the full clinical impact of VNS^11^ as even a seemingly modest frequency reduction can translate into substantial improvements in seizure burden and overall quality of life.

The diverse range of epilepsy etiologies and syndromes in our study expands our understanding of VNS applicability. Patients with LGS showed a response rate of approximately 56%, consistent with previous reports.^7^ VNS offers a significant nonpharmacological option for managing this complex condition, which includes various seizure types and considerable cognitive impairment, serving as an alternative to dietary therapy or epilepsy surgery. While approximately half of LGS patients benefit from VNS,^7^ our findings indicate a reduced rate of medication reduction in this cohort, underscoring the refractory nature of LGS and the necessity for ongoing polytherapy in numerous instances. Conversely, both patients with Dravet syndrome in our study exhibited remarkable responses. The limited sample size is nonetheless encouraging, considering the refractory nature of Dravet syndrome. This aligns with a meta‐analysis reporting a 52.9% responder rate in Dravet syndrome.^8^ We found a reduced medication decrease rate in this group, indicating that LGS is very resistant and requires polytherapy in many instances. However, both Dravet syndrome patients in our study responded well. While our finding in two patients with Dravet syndrome is anecdotal, it is consistent with recent meta‐analyses showing a responder rate of approximately 51–54% in this population, suggesting VNS is a viable option.^12^

Early VNS implantation is strongly correlated with better outcomes, reinforcing the importance of early intervention in refractory epilepsy and suggesting that prompt adjunctive therapy may limit neuronal damage and network reorganization caused by chronic, uncontrolled seizures. Clinical data reveal better responses in younger patients or those with shorter epilepsy histories, which supports the modest negative connection between pre‐VNS epilepsy duration and seizure reduction.^13^ Progressive epileptogenesis in persistent seizures may make neural network modulation harder over time. Early VNS intervention may impede this process or treat seizures before network remodeling. Our findings suggest exploring VNS early in the treatment course for children with treatment‐resistant epilepsy rather than after years of ineffective therapy. Timely intervention can preserve cognitive function and reduce the cumulative load of severe seizures, improving long‐term outcomes.

### 4.2 Pubertal hormonal influences

This study also sheds light on puberty and VNS therapy in adolescent girls with epilepsy. Most female patients in our group had seizures worsen around menarche. This shows how pubertal hormonal changes, ovulatory cycles and estrogen surges, affect seizure activity,^3^ generating a dynamic hormonal interplay that can compromise seizure control during puberty. This supports catamenial epilepsy, where seizure clusters coincide with menstrual cycle stages with high estrogen and low progesterone.^14^ As their bodies adjust to hormone changes, many adolescent girls with epilepsy have breakthrough seizures or more frequent seizures during puberty. This suggests that clinicians managing adolescent females with VNS must anticipate this period of vulnerability and consider proactive strategies, such as cyclical adjunctive therapy with acetazolamide or benzodiazepines, as is common practice in catamenial epilepsy management.^15^

Interestingly, active VNS therapy did not prevent this pubertal seizure aggravation. Seizures increased temporarily for all seven females in our sample who had VNS implanted before or during menarche. This shows that while VNS has neuromodulatory benefits, hormonal cues may still have a strong effect on brain excitability. Clinicians must anticipate seizure deterioration around menarche in adolescent girls, even those undergoing VNS therapy. Medication modifications and VNS output can reduce this risk.

While some literature suggests that puberty does not uniformly worsen epilepsy, with some patients experiencing improvements or remission, particularly in certain idiopathic epilepsies,^16^ and even fewer seizures after menarche in some female cohorts,^17^ our findings show a clear trend toward seizure exacerbation in this treatment‐resistant population They may be more sensitive to hormone changes because of their severe epilepsy. The findings emphasize the importance of hormonal factors in epilepsy management in adolescent girls. Glutamatergic activity and seizure focus irritability may be enhanced by estrogen,^18^, ^19^ as well as medication metabolic alterations associated with puberty,^2^, ^20^ such as growth spurts increasing the volume of distribution. Thus, epilepsy management is complicated by puberty.

Our data suggest that clinicians should be aware of seizure surges around menarche, especially in girls receiving VNS, and create preemptive management options such as medication changes, benzodiazepine rescue medicine, or enhanced VNS stimulation settings. Our findings also raise crucial considerations about the optimal timing of VNS implantation relative to puberty. Despite VNS, our sample, with a median implantation age of 9, exhibited pubertal seizure aggravation. Earlier implantation may attenuate this effect. Prospective studies on hormone levels, VNS output, and seizure frequency in pubertal patients are needed to improve treatment during this key developmental phase.

### 4.3 Clinical implications of magnet use and seizure severity

Our study shows that magnet response predicts pediatric epilepsy VNS therapy. Patients who eliminated or reduced seizures with the magnet were more likely to attain overall responder status (≥ 50% seizure reduction), supporting prior findings. Active magnet use (with the device on) versus sham (magnet off) revealed a trend toward seizure cessation, but responders had magnet success.^7^ Success with magnets within 6–12 months significantly predicts a positive response to VNS. Loss of magnet response does not prevent a beneficial outcome but reduces the likelihood.^7^ Furthermore, frequent and successful magnet use may itself contribute to the cumulative therapeutic dose of stimulation over time, enhancing long‐term outcomes.

Mechanistically, the magnet instantly stimulates neuromodulation upon seizure start. Effective magnet use suggests the epileptic network is sensitive to vagal input and receptive to VNS‐mediated noradrenergic or other routes. However, inadequate magnet use may imply circuits less impacted by the vagus or rapid seizure propagation beyond VNS intervention.^7^ Understanding and using the magnet are also important, with younger children or those with drop attacks exhibiting “no benefit” due to deployment issues.^7^

In nearly three‐quarters of patients, seizure intensity improved beyond frequency. These included decreased seizure violence, shorter duration, milder dips, and faster recovery. Although seizure frequency frequently decreases, some patients with < 50% frequency reduction show milder episodes. VNS's neuromodulatory effects on subcortical arousal systems^21^ may lessen seizure severity in “non‐responders”.^7^ Clinicians should evaluate VNS efficacy based on seizure frequency and intensity, as seizure quality improves patients' everyday lives.

### 4.4 Impact on status epilepticus and medication management

In this study, VNS implantation significantly reduced status epilepticus. While retrospective, the reduction from two‐thirds of patients experiencing status epilepticus pre‐VNS to one‐sixth post‐VNS implies VNS may prevent or reduce prolonged seizures. This aligns with case series showing VNS success in super‐refractory status epilepticus.^22^ Raising the seizure threshold and inhibiting aberrant discharges may prevent seizure generalization into status epilepticus.^23^, ^24^ Post‐VNS medication adherence and polypharmacy reduction may help. It appears that post‐VNS status epilepticus was almost exclusively detected in non‐responders, indicating that VNS protects individuals with effective seizure control. VNS may comfort families by protecting them from catastrophic seizures.

Over half of our patients reduced ASM with VNS, often by stopping enzyme‐inducing medications or benzodiazepines, enhancing alertness, and minimizing drug interactions. This supports simplifying polytherapy during adulthood.^22^ LGS patients had less frequent drug decreases, reflecting their complex prescription regimens, but our findings recommend cautious medication tapering in responsive individuals to reduce pill load and enhance adherence. Post‐VNS ASM care requires seizure control and medication toxicity reduction.

EEG abnormalities may be linked to etiology, according to our results. In structurally disordered brains, VNS modulates epileptiform networks more preferentially, as interictal EEG improved more in these patients.^25^ In contrast to genetically driven epilepsies, EEG improvement in responsive structural epilepsy patients may indicate network stabilization.^26^, ^27^, ^28^, ^29^

### 4.5 Mechanistic considerations

VNS exerts its antiepileptic effects through complex neuromodulatory mechanisms involving vagal afferents, the nucleus tractus solitarius, and projections to the locus coeruleus (LC) and raphe nuclei, releasing norepinephrine and serotonin, respectively.^30^, ^31^, ^32^, ^33^ Norepinephrine from the LC enhances cortical inhibition and desynchronizes epileptic networks, raising the seizure threshold.^31^, ^32^, ^34^ This broad neuromodulation likely explains VNS's efficacy across diverse seizure types, potentially explaining the progressive improvement in seizure control often observed over time. The superior outcomes with earlier VNS implantation may reflect the developing brain's greater plasticity.^35^

Magnet‐triggered pulses offer insight into acute mechanisms: a sudden vagal input surge likely halts seizure propagation, potentially by interrupting thalamocortical synchronization or inhibiting aberrant firing.^7^, ^36^ A study showed that magnet use effectively aborted seizures in 67% of patients, many becoming long‐term responders,^7^ suggesting the susceptibility of certain seizures to timely vagal stimulation. In our cohort, magnet success was primarily observed in focal‐onset or secondarily generalized seizures. EEG improvements observed in some responders suggest VNS dampens underlying epileptic activity.^37^ While clinical seizure control can occur without noticeable EEG changes, EEG improvement provides objective evidence of VNS‐mediated modulation of pathological activity.^37^ Finally, hormonal influences warrant consideration. Estrogen's influence on neuronal excitability may temporarily counteract VNS's stabilizing effects.^3^ Adjunctive hormonal modulation therapies, such as acetazolamide or clobazam, or hormonal contraception, may prove beneficial, particularly during periods of significant hormonal fluctuation, as suggested by our findings in adolescent girls experiencing seizure exacerbation at menarche.

### 4.6 Limitations and future directions

This study has several important limitations. First, its retrospective, single‐center design and small sample size (*n* = 30) limit the generalizability of our findings and the precision of our statistical estimates, as evidenced by the wide confidence intervals for some odds ratios. A multivariate analysis to control for confounders was not feasible due to the limited sample size. Second, key outcomes such as seizure severity and magnet response were based on caregiver reports, which are subject to recall bias. While clinically meaningful, future prospective studies should incorporate standardized seizure diaries and quality‐of‐life instruments. Third, we did not systematically collect data on the use of the AutoStim feature, which is a potential confounder for both magnet use and overall efficacy. Fourth, the wide confidence interval for the menarche‐associated worsening indicates significant statistical imprecision, and this finding should be interpreted cautiously as a preliminary observation requiring further investigation. Finally, our study did not include systematic hormonal tracking or formal neuropsychological assessments, which would be valuable additions for future prospective research to objectively quantify the cognitive and endocrine impacts of VNS during adolescence.

Despite the small sample size and dependence on clinical records, this retrospective single‐center study provides useful insights into VNS therapy in pediatrics. While therapeutically meaningful, caregiver‐reported outcomes are subjective, but objective data were used to verify them. Pre‐ and post‐VNS seizure diaries and cognitive tests were not available, adding to the limitations. Despite these limitations, the study gives a complete summary of VNS outcomes, focusing on sex, condition, and puberty for subtle insights beyond responder/non‐responder ratings. Important findings include puberty's impact, magnet use as a biomarker, and status epilepticus prophylaxis. Future prospective research should examine hormonal effects on VNS efficacy, conduct randomized controlled trials in specific epilepsy subgroups, and evaluate biomarkers like heart rate variability and early post‐implantation EEG alterations. Further studies should examine how VNS affects child cognitive development and quality of life.

## 5 CONCLUSION

This study demonstrates that VNS benefits treatment‐resistant epileptic children and adolescents. Most patients' seizure frequency and severity decreased with VNS, simplifying treatment regimes. VNS reduced status epilepticus risk and enhanced EEG patterns. The advantages were seen across epilepsy types and severity. The study emphasizes preventive care during adolescence, when seizures may intensify even with VNS. The VNS magnet's ability to stop seizures highly predicted long‐term effectiveness, underlining the need for patient and family education. VNS affects the brain instantly (magnet use) and over time (long‐term seizure control and EEG alterations). To improve patient outcomes, this study recommends early VNS consideration, cautious monitoring during puberty, constant magnet use, and regular medication evaluation for pediatric epilepsy.

## AUTHOR CONTRIBUTIONS

**Jonadab dos Santos Silva:** Conceptualization (lead); methodology (lead); formal analysis (lead); writing—original draft (lead); writing—review and editing (equal). **Shaylla Villas Boas:** Conceptualization (supporting); investigation (lead); data curation (lead); validation (supporting); writing—review and editing (equal). **Henrique Januzzelli Pires do Prado:** Investigation (supporting); data curation (supporting); visualization (lead); methodology (supporting); writing—review and editing (equal). **Daniela Fontes Bezerra:** Investigation (supporting); data curation (supporting); resources (lead); project administration (supporting); validation (supporting); writing—review and editing (supporting). **Isabella D’Andrea Meira:** Supervision (lead); conceptualization (supporting); investigation (supporting); data curation (supporting); writing—review and editing (equal).

## FUNDING INFORMATION

This study did not receive any funding.

## CONFLICT OF INTEREST STATEMENT

None of the authors has any conflict of interest to disclose.

## STROBE STATEMENT

The authors have read the STROBE checklist for observational studies, and the article was prepared and revised accordingly.

**Test yourself**

1. Approximately what proportion of pediatric patients with treatment‐resistant epilepsy achieved a ≥50% reduction in seizure frequency at one year after VNS implantation in this study?
  - 25%
  - 45%
  - 67%
  - 80%
  - 90%
2. How did vagus nerve stimulation therapy affect the occurrence of status epilepticus in this pediatric cohort?
  - It had no effect on the frequency of status epilepticus
  - It increased the incidence of status epilepticus after implantation
  - It significantly reduced status epilepticus events (from approximately two‐thirds of patients pre‐VNS to about one‐sixth post‐VNS)
  - It reduced status epilepticus occurrence only in patients older than 12 years
  - It eliminated status epilepticus in all patients who had experienced it prior to implantation
3. What was observed regarding antiseizure medication use after one year of VNS therapy in these patients?
  - All patients were able to discontinue antiseizure medications due to VNS
  - Most patients needed higher doses or additional medications after VNS
  - Over half of the patients reduced the number of antiseizure medications following VNS implantation
  - VNS had no impact on the number or dose of medications required
  - Only patients with Lennox‐Gastaut syndrome were able to reduce their medications
4. Which of the following was identified as a strong predictor of positive VNS response (≥50% seizure reduction) in this study?
  - The specific epilepsy syndrome or etiology (e.g., genetic versus structural cause)
  - Successful use of the VNS magnet to acutely interrupt or shorten seizures
  - Patient sex (female versus male)
  - Higher number of antiseizure medications tried prior to VNS (polytherapy)
  - Presence of intellectual disability
5. Approximately what proportion of patients experienced an acute seizure interruption or reduction when the VNS magnet was swiped at seizure onset?
  - Around 10% of patients
  - Around 43% of patients
  - Around 60% of patients
  - Around 75% of patients
  - Nearly 90% of patients
6. In female patients receiving VNS, what impact did menarche (pubertal onset) have on seizure control as reported by this study?
  - VNS completely prevented any increase in seizures around menarche
  - The majority of girls had a transient worsening of seizures around the time of menarche despite VNS therapy
  - Seizure frequency uniformly improved around menarche in VNS‐treated females
  - Girls who received VNS before puberty had significantly worse long‐term seizure outcomes than those implanted after menarche
  - There was no relationship observed between menarche and seizure patterns in this cohort
7. What did the study find regarding the timing of VNS implantation in relation to epilepsy duration and seizure outcomes?
  - Later implantation after many years of epilepsy was associated with better seizure control
  - Shorter epilepsy duration before VNS implantation was correlated with greater seizure reduction
  - Duration of epilepsy prior to VNS showed no correlation with seizure outcome
  - VNS was only effective if implanted within the first year of epilepsy diagnosis
  - Patients with more than 15 years of epilepsy showed the best response to VNS
8. Which statement about electroencephalography (EEG) changes during VNS therapy is correct according to this study's findings?
  - All patients showed significant EEG improvements with VNS therapy
  - About 23% of patients showed improved EEG findings (reduced epileptiform activity or normalized background), mainly among male patients and pre‐pubescent girls
  - EEG improvements occurred exclusively in patients who became completely seizure‐free
  - All post‐pubertal female patients showed marked EEG normalization after VNS implantation
  - EEG findings worsened in most patients following VNS implantation
9. Based on the study's findings, what proactive management strategy should clinicians consider for adolescent girls with VNS as they approach puberty?
  - No changes are necessary because VNS will prevent hormonally driven seizure exacerbations
  - Anticipate potential seizure worsening around menarche and adjust therapy (e.g., optimize medications, consider rescue benzodiazepines, or adjust VNS settings) in advance
  - Discontinue VNS before puberty to avoid interference from hormonal changes
  - Rely solely on increased magnet use during menses to control catamenial seizures without other adjustments
  - Delay any medication adjustments until after complete pubertal development
10. Which of the following best describes VNS efficacy across different epilepsy etiologies and syndromes in this pediatric cohort?
  - VNS was effective only in patients with Lennox‐Gastaut syndrome and not in other epilepsy syndromes
  - Structural (lesional) epilepsies responded to VNS, whereas genetic epilepsies showed no benefit
  - The epilepsy etiology strongly determined VNS outcome, with certain causes consistently predicting non‐response
  - VNS demonstrated potential benefits across a wide range of etiologies and syndromes, with no single epilepsy type categorically precluding a response
  - Only patients with unknown etiology responded favorably to VNS

*Answers may be found in the* *supporting information*.

## Supporting information


Data S1.

## Data Availability

The data that support the findings of this study are available from the corresponding author upon reasonable request.
