# Supplementary material for: Efficacy of vagus nerve stimulator during transition to adulthood in patients with treatment‐resistant epilepsy
Source: Epileptic Disord. 2026 Jan 19;28(1):142–54. doi: 10.1002/epd2.70133 (PMC12964184; doi:10.1002/epd2.70133)
Supplement: Supplementary file 1 — Data S1. [file EPD2-28-142-s001.docx]

Q1. **Answer: C**

**Rationale:** The study found that 20 of 30 patients (66.7%, approximately 67%) achieved ≥50% seizure reduction at last follow-up. Answer A (25%) is too low and does not reflect the study's favorable outcomes. Answer B (45%) underestimates the responder rate. Answers D (80%) and E (90%) overestimate the proportion of responders; while outcomes were positive, not all patients achieved this level of seizure control.

Q2. **Answer: C**

**Rationale:** Status epilepticus incidence fell from 67% pre-VNS to 17% post-VNS (p = 0.024), representing a significant reduction from roughly two-thirds to one-sixth of patients. Answer A is incorrect because VNS did have a statistically significant effect. Answer B is incorrect because VNS reduced, not increased, status epilepticus. Answer D is incorrect because the reduction was observed across ages, not limited to those over 12 years. Answer E overstates the benefit; while substantially reduced, status epilepticus was not completely eliminated in all previously affected patients.

Q3. **Answer: C**

**Rationale:** The study found that 17 patients (56.7%) reduced their antiseizure medication count after VNS, with the median number decreasing from 4 to 3. Answer A is incorrect because complete discontinuation was not achieved. Answer B is incorrect because most patients reduced rather than increased medications. Answer D is incorrect because VNS did impact medication burden. Answer E is incorrect because medication reduction occurred across different etiologies, not exclusively in Lennox-Gastaut syndrome; in fact, LGS patients had less frequent drug reduction due to their condition's complexity.

Q4. **Answer: B**

**Rationale:** The study demonstrated that patients who achieved acute seizure interruption or reduction with the VNS magnet were significantly more likely to attain overall responder status, with 12 of 13 magnet responders (92.3%) achieving ≥50% seizure reduction. Answer A is incorrect because no etiology or syndrome categorically predicted response. Answer C is incorrect because patient sex did not significantly predict VNS response. Answer D is incorrect because the number of prior medications was not identified as a predictor. Answer E is incorrect because intellectual disability was not reported as a predictor of response.

Q5. **Answer: B**

**Rationale:** The study found that 13 patients (43.3%) had seizures terminated or reduced in severity when the magnet was swiped at seizure onset. Answer A (10%) significantly underestimates the magnet efficacy rate. Answers C (60%), D (75%), and E (90%) overestimate the proportion of patients who benefited from acute magnet activation; while magnet response was a strong predictor of overall VNS success, it was effective in approximately 43% of the cohort.

Q6. **Answer: B**

**Rationale:** All seven females who had VNS implanted before or during menarche experienced transient seizure worsening around puberty, indicating that active VNS therapy did not prevent pubertal seizure aggravation. Answer A is incorrect because VNS did not prevent perimenstrual seizure increases. Answer C is incorrect because seizures worsened, not improved, around menarche. Answer D is incorrect because the study did not show worse long-term outcomes for those implanted before puberty. Answer E is incorrect because a clear relationship between menarche and seizure exacerbation was observed.

Q7. **Answer: B**

**Rationale:** The study showed that for each additional year of uncontrolled epilepsy before VNS implantation, the odds of being in a lower response category increased by 22% (OR 1.22, 95% CI 1.01–1.47, p = 0.034), meaning earlier intervention was associated with better outcomes. Answer A is incorrect because longer duration was associated with worse, not better, outcomes. Answer C is incorrect because a statistically significant correlation was found. Answer D is incorrect because VNS was effective beyond the first year of diagnosis. Answer E is incorrect because longer epilepsy duration predicted poorer response.

Q8. **Answer: B**

**Rationale:** Seven of 30 patients (23.3%) exhibited EEG improvement after VNS therapy, and all seven were either male or pre-pubescent females. Answer A is incorrect because only a subset, not all patients, showed EEG improvement. Answer C is incorrect because EEG improvement occurred in responders who did not necessarily achieve complete seizure freedom. Answer D is incorrect because no post-menarche females showed EEG improvement; in fact, this group had consistently abnormal EEGs. Answer E is incorrect because EEG findings generally remained stable or improved, not worsened.

Q9. **Answer: B**

**Rationale:** The study demonstrated that all seven females with VNS implanted before or during menarche experienced transient seizure worsening around puberty. The authors recommend that clinicians anticipate seizure surges around menarche and develop preemptive management strategies including medication optimization, rescue benzodiazepines, or enhanced VNS stimulation settings. Answer A is incorrect because VNS did not prevent perimenstrual seizure worsening. Answer C is incorrect because discontinuing VNS is not recommended. Answer D is incorrect because a multimodal approach is preferred over magnet use alone. Answer E is incorrect because proactive, not delayed, management is recommended.

Q10. **Answer: D**

**Rationale:** The study found that VNS was effective across different epilepsy etiologies, with no significant differences in responder rates by etiology. Structural etiologies showed a responder rate of 78.6% compared to 61.1% in non-structural etiologies, but importantly, no etiology or syndrome categorically precluded a response. Answer A is incorrect because VNS efficacy extended beyond Lennox-Gastaut syndrome. Answer B is incorrect because both structural and genetic epilepsies could respond to VNS. Answer C is incorrect because etiology did not strongly determine outcome. Answer E is incorrect because response was observed across all etiological categories, not exclusively in those with unknown etiology.
